# The School Clinic

**Published:** 1911-10-14

**Authors:** 


### THE SCHOOL CLINIC.

### I.—Why School Clinics are Necessary.

### SOME GENERAL CONSIDERATIONS.

School Clinics have in a few places already been established, and the advisability of some such method being widely adopted in connection with the treatment of school children will become more and more prominent. The extent of abnormal conditions in school children throughout the country is now fairly accurately known. How far the children have profited by the notification of ailments is not perhaps so accurately known, though no doubt endeavours are being made to gain some knowledge upon the point. It would materially aid consideration of the subject if reliable statistics were obtainable, not only from London and the various provincial towns, large and small, but also from the country districts. Such statistics would be additionally valuable if it could be ascertained from what source treatment had been obtained. The percentage of cases of treatment will no doubt vary considerably in different localities. Where charitable institutions are accessible the number of cases treated will probably be higher than in districts not provided with such institutions. It may not follow, possibly, that when a child is taken from a village to a hospital in a neighbouring town that the treatment required will be given. Allowing, however, that outside London, as within it, the presence of a charitable institution does not necessarily ensure efficient treatment, there will probably be a certain number of cases obtaining treatment at such an institution which would otherwise go untreated.

In considering statistics with regard to the number of cases of school children treated for various ailments, not only must regard be paid to locality and the facilities of the locality, but the opinions of the medical inspector may require to be taken into account. One inspector may hold very different views from another as to the necessity for treatment. The temperament of one may incline him to "magnify his office" by an important show of necessitous cases as the result of his visits. Another having always before him the difficulties of treatment, may seek to reduce his recommendations to a minimum. An illustration of the results of the opinion of the more energetic adviser may be given. A medical inspector prodigal in his recommendations was especially so as regards operation for adenoids. Some children were taken to accessible hospitals seventeen or eighteen miles distant, and the parents returned—annoyed at having wasted railway fares—with the opinion given at hospitals that no operation was necessary. Other children were taken to local practitioners by whom, in some instances, refusal to operate was made, while others—more good-natured—performed the operation, but afterwards laughingly commented upon the lack of need for it. There is, in a condition like that of adenoids, considerable room for difference of opinion as to the indications for operative treatment, and opinion in such matters must largely affect the value of statistics as to the results of inspection.

In this connection another point is worthy of consideration. When tabulating results the recording of the fact that some treatment has been given need not imply very much. There is treatment and there is efficient treatment. In spite, however, of the difficulties which surround the collecting of statistics bearing upon treatment, a record of results should be of considerable value.

Cases requiring treatment may perhaps be divided into two classes. Firstly, those in which treatment is necessary; secondly, those in which it is perhaps advisable but cannot be said to be absolutely necessary. It is possible that statistics would show that the number of cases left untreated which belong to the first class is not so great as might be supposed. However that may be, since the aim of medical inspection is practical benefit to the children, obviously it is essential to get as near the truth as possible with regard to treatment. If we take a favourable view and allow, in spite of many pessimistic statements to the contrary, that the number of cases of school children left untreated which require treatment is not large, we cannot doubt that there must always be a certain number which will require in some form or another the attention of the central organisation.

Before embarking upon any new departure it is necessary to consider how far existing means of dealing with the matter are inadequate. The means at present available are the local practitioner, the Poor Law, and charitable institutions. The local practitioner has without doubt played his part in the treatment of school children, but there are certain cases which not many medical men in general practice are in a position to deal with, and there are others which, although of a nature they are accustomed to treat, they cannot be expected to undertake without remuneration. Medical men in the service of the Poor Law are in a somewhat similar position. Some cases the Poor Law medical officer will probably not be fitted to treat, and the treatment of others he will not consider to be a part of his duties unless some fresh arrangements are made by him with the Guardians. There remain the charitable institutions. How far are they adequate, or inadequate, to deal with the treatment of school children? Objection to the use of hospitals may be made on many grounds. Some object on moral grounds. Dr. Snell, of Coventry, for example, says that his Education Authority object to the "painfully pernicious system" of referring children early in life to charities. The principle "that anything worth having is worth paying for" no doubt is good when it can be paid for, but unfortunately in many cases payment can only be made at great sacrifice or may be virtually impossible. There are cases, too, with regard to which the parents very reluctantly take the advice given, and it must be admitted that in some instances, as has previously been indicated, the parents may be justified in being reluctant to act upon the advice of a medical inspector. If parents are virtually going to be forced to get their children treated and be forced also to pay for the treatment, the opinion of the medical inspector will need to be of considerable value. Whether, however, the opinion of the medical inspector is, or is not to be, the sole authority, few probably will maintain strongly that hospital advice and treatment are wholly pernicious. Yet the value of hospitals may be considered to be found wanting on other grounds than those connected with their position as charities. In spite of the facilities they afford for dealing with a considerable number of cases, in large towns it is extremely difficult, if not impossible, to treat satisfactorily more than a comparatively small part of the children notified in the schools as possessing some ailment. Cases such as those requiring to be tested for spectacles or an operation for adenoids are more especially referred to. There are other cases, again, such as discharging ears, which may require daily attention, and this is rarely given in such cases at hospitals. Failure to obtain the required attention may occur not only at hospitals in large towns, but in cases visiting provincial hospitals from neighbouring villages. For example, a child badly affected with adenoids was taken at intervals during the space of three months from a village to a hospital in a neighbouring town without any operation being performed. Money was paid for several railway fares to obtain medicine only. It may be argued that the money paid in railway fares should have been given to a local doctor for an operation. In this instance, however, the local doctor disliked to undertake, if indeed he ever did undertake, the operation. Again an official use of hospitals, even if restricted to the children of those parents who cannot afford to pay for treatment, raises questions besides those of the power of the hospitals to deal with a large addition to the present number of their patients. The honorary medical officers may object to work for the State without remuneration, not only for their own sake but for the sake of their colleagues who are not connected with the hospitals. Apparently in consideration of the attitude of some medical men the largest Education Authority arranged with some hospitals for the use of out-patient rooms on certain days and paid junior medical men to treat cases sent from the schools. There have been many objections to this system. Some hospitals refused to entertain the idea, and some others which undertook to follow out the suggestion for a time have since abandoned it. In a large provincial town also, apparently an arrangement between the hospitals and the Education Authority has broken down. It seems clear, therefore, that any permanent arrangement should be made with the hospitals by means of which school children will be treated, appears to be extremely improbable. But allowing that some system of treatment entirely independent of hospitals will before long be established, another question arises. Are the hospitals to cease entirely to perform any office for children found to be defective in the schools? With regard to cases requiring admission this can scarcely be so. Operations for radical cure of hernia, orthopædic operations, removal of masses of tuberculous glands, and many other surgical procedures will need to be carried on within hospital walls. So far as out-patients are concerned, the establishing of school clinics would probably deprive hospitals of all children recommended for treatment in schools. Yet in some instances they might be advised to go to hospitals for an opinion. In several quarters recently it has been suggested that the out-patient departments of hospitals should be mainly consultative clinics. As consultative clinics hospitals might still play a useful part in dealing with school children.

Since the hospitals probably will not in the future occupy a large place in the treatment of school children, other methods for getting practical results from medical inspection require to be considered. Although the medical man in general practice has without doubt aided in obtaining some practical result, there are some cases, such as the testing for defective eyesight and attention to decayed teeth, which can scarcely be dealt with elsewhere than at some central institution. The operation for adenoids also, although no doubt it can be performed in a doctor's surgery, is much more easily carried out in some place designed to deal with a considerable number of such cases. It does not necessarily follow that the establishing of such an institution or school clinic would exclude medical practitioners from taking part in the work. A staff of medical men in general practice would complicate the organisation and give some advantage possibly to a favoured few, but it might possess features which would compensate for difficulties. A matter like this is one which would probably give rise to a considerable amount of discussion. Dr. Lewis Williams, of Bradford, where a school clinic has been established, considers that from the point of view of the medical man in practice it is better that a whole-time officer should treat the children than that the work should be done by a few medical practitioners to the possible disadvantage of others. Dr. Priestley introduces another aspect of the question when he expresses the view that treatment should be in other hands than in the hands of those whose duties are to inspect the school children. He considers that outside opinions are an advantage. It cannot be doubted that the aspects of affections of children seen in school are circumscribed, and without some stimulating external influences the medical man who deals only with school children would tend to drift into perfunctory methods of work. In the early days of the establishing of clinics and while some particular clinic is possibly an object of interest to many outside the Education Authority under whose auspices it has been established, every detail of the work may be carried out with something perhaps approaching to enthusiasm. Should, however, there be school clinics everywhere and all officered by men confined entirely to school work, it is to be feared that the character of the work would not be maintained at a high level.

